# Physical Mapping of the 5S and 18S rDNA in Ten Species of *Hypostomus* Lacépède 1803 (Siluriformes: Loricariidae): Evolutionary Tendencies in the Genus

**DOI:** 10.1155/2014/943825

**Published:** 2014-10-27

**Authors:** Vanessa Bueno, Paulo César Venere, Jocicléia Thums Konerat, Cláudio Henrique Zawadzki, Marcelo Ricardo Vicari, Vladimir Pavan Margarido

**Affiliations:** ^1^Universidade Tecnológica Federal do Paraná, Prolongamento da Rua Cerejeira s/n, São Luiz, 85892-00l Santa Helena, PR, Brazil; ^2^Departamento de Biologia e Zoologia, Universidade Federal de Mato Grosso, Instituto de Biociências, Avenida Fernando Correa da Costa s/n, Coxipó, 78060-900 Cuiabá, MT, Brazil; ^3^Universidade Estadual do Oeste do Paraná, Centro de Ciências Biológicas e da Saúde, Rua Universitária 2069, 85801-110 Cascavel, PR, Brazil; ^4^Departamento de Biologia/Nupélia, Universidade Estadual de Maringá, Avenida Colombo 5790, 87020-900 Maringá, PR, Brazil; ^5^Departamento de Biologia Estrutural, Molecular e Genética, Universidade Estadual de Ponta Grossa, Avenida Carlos Cavalcanti 4748, 84030-900 Ponta Grossa, PR, Brazil

## Abstract

*Hypostomus* is a diverse group with unclear aspects regarding its biology, including the mechanisms that led to chromosome diversification within the group. Fluorescence *in situ* hybridization (FISH) with 5S and 18S rDNA probes was performed on ten Hypostomini species. *Hypostomus faveolus*, *H. cochliodon*, *H. albopunctatus*, *H.* aff. *paulinus,* and *H. topavae* had only one chromosome pair with 18S rDNA sites, while *H. ancistroides*, *H. commersoni*, *H. hermanni*, *H. regani,* and *H. strigaticeps* had multiple 18S rDNA sites. Regarding the 5S rDNA genes, *H. ancistroides*, *H. regani*, *H. albopunctatus*, *H.* aff. *paulinus,* and *H. topavae* had 5S rDNA sites on only one chromosome pair and *H. faveolus*, *H. cochliodon*, *H. commersoni*, *H. hermanni,* and *H. strigaticeps* had multiple 5S rDNA sites. Most species had 18S rDNA sites in the telomeric region of the chromosomes. All species but *H. cochliodon* had 5S rDNA in the centromeric/pericentromeric region of one metacentric pair. Obtained results are discussed based on existent phylogenies for the genus, with comments on possible dispersion mechanisms to justify the variability of the rDNA sites in *Hypostomus*.

## 1. Introduction

Loricariidae is a species-rich and diverse family, distributed through Central and South America [1, 2]. It is composed of seven subfamilies: Delturinae, Hypoptomatinae, Hypostominae, Lithogeneinae, Loricariinae, Neoplecostominae, and Otothyrinae [1, 3–5]. The Hypostominae contain a great number of nominal species with unclear status, and the systematics of the subfamily is not well resolved [3]. Armbruster [1] proposed the division of the subfamily in five tribes, namely, Corymbophanini, Rhinelepini, Hypostomini, Pterygoplichthini, and Ancistrini. The only genus recognized for Hypostomini would be* Hypostomus*, with* Aphanotorulus*,* Cochliodon*,* Isorineloricaria*,* Squaliforma,* and* Watwata *as synonyms. However, molecular studies based on mitochondrial rRNA gene sequences,* D-loop,* and ITS sequences indicated relevant distinctions among* Squaliforma*,* Isorineloricaria*,* Aphanotorulus,* and* Hypostomus*, therefore not supporting the synonymization of these genera with* Hypostomus *[6, 7]. Further analyses have not been performed on the Hypostomini to verify which genera should be recognized, and there seems to be no consensus about which phylogeny should be adopted for* Hypostomus*, with a number of further studies about other aspects of the genera considering each hypothesis as valid.

Karyotypic studies of* Hypostomus *initiated with the analysis of* Hypostomus plecostomus *(Linnaeus 1758) [8]. The diploid number found, 2*n* = 54 chromosomes, is the lower diploid number observed for the genus. Further studies showed diploid numbers ranging from 2*n* = 64 to 2*n* = 84 chromosomes [9, 10], indicating that the specimens analyzed by Muramoto et al. [8] might have been misidentified [9]. The number of species cytogenetically analyzed is increasing, but is still far from representing a significant portion of the genus [11]. Also, most studies discuss only the diploid number and location of the AgNORs, ignoring important markers as heterochromatin distribution and mapping of rDNAs sites.

Few species of* Hypostomus* had their rDNA sites mapped. The lack of information on the number and location of the 5S and 18S rDNA sites in* Hypostomus *hinders a broad comparative analysis for the genus. Since the relationships among species of* Hypostomus *are still unclear, the present study aims to perform the mapping of 5S and 18S rDNA of ten species of* Hypostomus* comparing the results with available data for this group to verify the existence of possible evolutionary trends on the genus regarding this trait.

## 2. Material and Methods

The specimens were captured from three distinct Brazilian localities: Piquiri River (municipality of Nova Laranjeiras, Paraná state, Upper Paraná River basin), Iguaçu River (municipality of Foz do Iguaçu, Paraná state, Middle Paraná River basin), and Taquaralzinho River (municipality of Barra do Garças, Mato Grosso state, Upper Araguaia River basins). The collection sites, number of males and females, and catalog numbers of voucher specimens, which were deposited in the Coleção Ictiológica do Núcleo de Pesquisas em Limnologia, Ictiologia e Aquicultura-Nupélia-Universidade Estadual de Maringá, Brazil, are summarized in Table 1. The specimens were anesthetized and sacrificed through clove-oil overdoses [13]. Metaphasic cells were obtained from the kidney through the air-drying technique [14]. Fluorescence* in situ* hybridization was performed according to Pinkel et al. [15], with modifications suggested by Margarido and Moreira-Filho [16]. The 18S rDNA probes were obtained according to Hatanaka and Galetti [17], and 5S rDNA probes were obtained according to Martins and Galetti Jr. [18]. The probes were marked through nick translation, with biotin-16-dUTP (18S rDNA) and digoxigenin-11-dUTP (5S rDNA) (Roche). Detection and amplification of the hybridization signals were performed with avidin-FITC and antiavidin biotin (Sigma-Aldrich) for the 18S rDNA probes, and antidigoxigenin rhodamine (Roche Applied Science) for the 5S rDNA probes. The slides were counterstained with DAPI and analyzed on the epifluorescence microscope Olympus BX 61. Thirty metaphases per individual were analyzed. Chromosomes were classified according to Levan et al. [19].

## 3. Results

**Figure 1:**
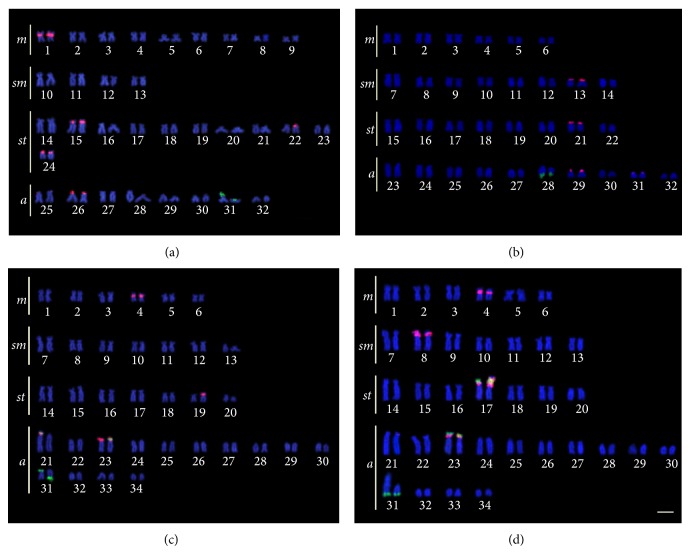
Double FISH karyotypes with 5S rDNA probes (rhodamine, red) and 18S rDNA probes (FITC, green) of (a)* Hypostomus faveolus*; (b)* Hypostomus cochliodon*; (c)* Hypostomus commersoni *from the Piquiri River and (d)* Hypostomus commersoni *from the Iguaçu River. The bar represents 5 *μ*m.

**Figure 2:**
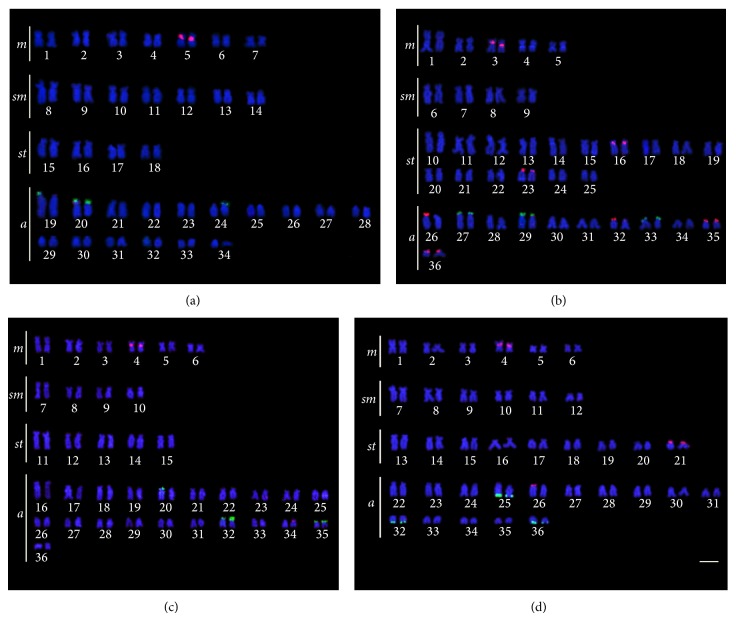
Double FISH karyotypes with 5S rDNA probes (rhodamine, red) and 18S rDNA probes (FITC, green) of (a)* Hypostomus ancistroides*; (b)* Hypostomus hermanni*; (c)* Hypostomus regani*; (d)* Hypostomus strigaticeps*. The bar represents 5 *μ*m.

**Figure 3:**
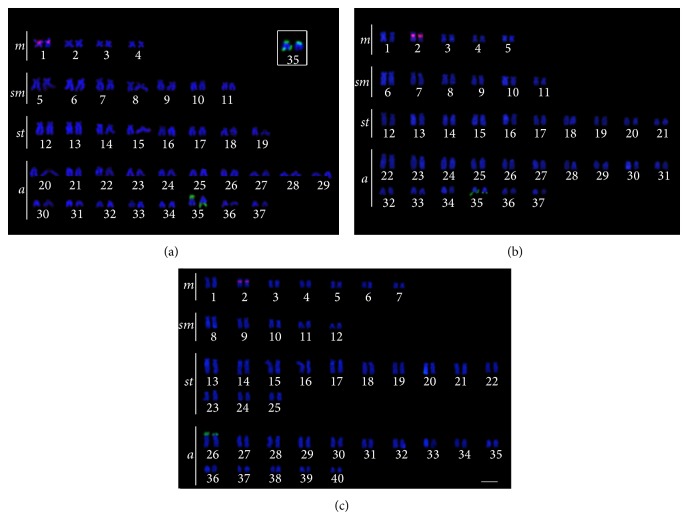
Double FISH karyotypes with 5S rDNA probes (rhodamine, red) and 18S rDNA probes (FITC, green) of (a)* Hypostomus albopunctatus*, with a variation of the position of the 18S rDNA sites evidenced on the box; (b)* Hypostomus *aff.* paulinus* and (c)* Hypostomus topavae*. The bar represents 5 *μ*m.

*Hypostomus faveolus *Zawadzki, Birindelli, and Lima, 2008,* H. cochliodon* Kner, 1854,* H. albopunctatus *(Regan, 1908),* H. *aff.* paulinus* (Ihering, 1905), and* H. topavae* (Godoy, 1969) had only one chromosome pair with 18S rDNA sites, while* H. ancistroides *(Ihering, 1911),* H. commersoni *Valenciennes, 1836,* H. hermanni *(Ihering, 1905),* H. regani* (Ihering, 1905), and* H. strigaticeps *(Regan, 1908) had multiple 18S rDNA sites. All species had 18S rDNA sites located in the telomeric region of the chromosomes. As for the 5S rDNA,* H. ancistroides*,* H. regani*,* H. albopunctatus*,* H. *aff.* paulinus,* and* H. topavae* had 5S rDNA sites on only one chromosome pair, and* H. faveolus*,* H. cochliodon*,* H. commersoni*,* H. hermanni,* and* H. strigaticeps *had multiple 5S rDNA sites (Figures 1, 2, and 3). The number and position of both 5S and 18S rDNA differed between the populations for* H. commersoni*, and both populations showed syntenic sites. The chromosome size difference in* H. commersoni *(pair 31) is due to a heterochromatic block that is amplified in just one homologous of the chromosome pair. Results are summarized in Table 2, along with the available data for localization of rDNA in* Hypostomus*.

## 4. Discussion

A single pair bearing 5S and 18S rDNA sites has been considered plesiomorphic for Loricariidae, given that this characteristic was observed in the outgroup Trichomycteridae and some other Loricariidae genera (*Neoplecostomus*,* Kronichthys*,* Isbrueckerichthys,* and* Parotocinclus*) considered phylogenetically basal through morphological analysis [1, 27]. Artoni and Bertollo [28] also consider single NORs as the ancestral phenotype for Loricariidae. These characteristics observed on basal genera for Loricariidae, besides other tribes of Hypostominae such as Pterygoplichthini and Ancistrini [23, 29], support the hypothesis that the presence of one site of 5S and 18S rDNAs is basal for* Hypostomus*.

**Figure 4:**
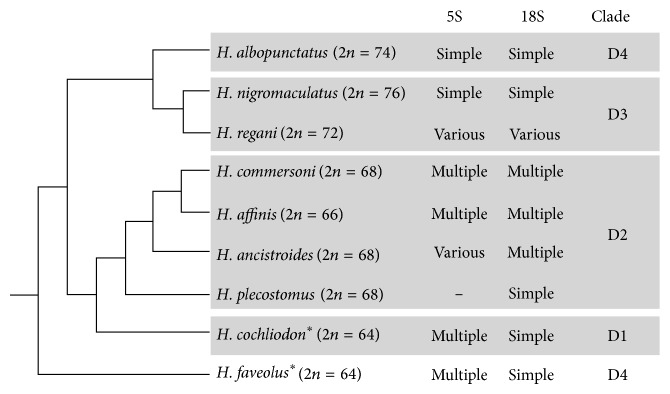
Data on rDNA sites number plotted against the phylogenetic relationship between species. The cladogram is only representative and is based on the phylogeny proposed by Montoya-Burgos (2003), with addition of species marked with an asterisk. “Clades” refer to the clades proposed by the same author.* Hypostomus cochliodon *is added considering information from Armbruster [1] and* H. faveolus *considering information from Martinez [12].

It is known that the diploid numbers of* Hypostomus *are likely correlated with their phylogeny and distribution [9]. The following discussion relies on this correlation and on existent phylogenies for the genus; therefore, the discussion will be organized according to diploid number ranges. The correlation among diploid numbers, data obtained from the present paper, and phylogenetic relationships of* Hypostomus *are simplified in Figure 4.* Hypostomus faveolus *and* H. cochliodon *are the species with the lower diploid numbers in* Hypostomus *that had the 5S and 18S rDNA location mapped. Both species had a single pair bearing 18S rDNA, although on different positions on the chromosome for each species, and multiple chromosomes bearing 5S rDNA. Considering the diploid number of 54 chromosomes basal for the family Loricariidae, lower diploid numbers would be considered basal for* Hypostomus *[30]. Also, a phylogenetic analysis performed by Martinez [12] with the sequences of mitochondrial cytochrome b and partial sequences of the 16S rRNA and nuclear F-reticulon 4 genes considered* H. faveolus *the most basal species for* Hypostomus*. The status of* H. faveolus *as a basal species is compatible with our results, since this species presented one 18S rDNA site, which corroborates the hypothesis that the presence of one 18S rDNA site is basal for* Hypostomus.* As for the 5S rDNA, it is not possible to determine whether multiple pairs bearing 5S rDNA sites are an ancestral condition for the genus or an apomorphy for the analyzed species.

The species with 2*n* = 68 and 2*n* = 72 chromosomes (*H. ancistroides*,* H. commersoni*,* H. hermanni*,* H. regani, *and* H. strigaticeps*) had multiple chromosomes bearing 18S rDNA sites. Most species also presented multiple chromosomes bearing 5S rDNA sites, with only* H. ancistroides *and* H. regani *with 5S rDNA on a single chromosome pair. There was variation on the location of some sites among the analyzed populations of* H. commersoni*. Only a small number of specimens of* H. commersoni *were collected; therefore it was not possible to verify how the rDNA is distributed within the populations. There is also a description of* H. regani *from Piumhi River (São Francisco River basin, state of São Paulo) with multiple pairs bearing 5S rDNA and a single pair bearing 18S rDNA [25]. It differs from that observed for the population from the Piquiri River (Paraná River basin, Paraná state) that showed a single pair bearing 5S rDNA and multiple pairs bearing 18S rDNA. Analyses of other populations of* H. ancistroides *(Paraná River basin, São Paulo state) [22] and* H. strigaticeps *(Paraná River basin, between Paraná and São Paulo states) [24] also showed different results from that observed on the present study, with multiple 5S rDNA sites for* H. ancistroides* and two pairs bearing 18S rDNA sites for* H. strigaticeps*. Considering the small number of species analyzed through* in situ *hybridization, there seems to be a relevant occurrence of variation on the number and position of the rDNA sites. Although this group includes species with similar diploid numbers (2*n* = 68 to 2*n* = 72) and number of 5S and 18S sites, these characteristics are not synapomorphic [1, 7, 12]. The variation on the number of rDNA sites, even among populations of the same species, and the existence of phylogenetic hypotheses that separate species with similar number and location of rDNA sites indicate that this characteristic might not be reliable to establish relationships within* Hypostomus*, but seems to have potential as a population marker.

The variation on the number and location of 18S rDNA sites which occurs even among individuals of the same species suggests the existence of dispersion mechanisms on these sites. The particular chromosome evolution of this genus through centric fissions and pericentric inversions [11, 30] may be partly responsible for this diversity in* Hypostomus*. However, it is known that some retrotransposons are specific for rDNA sequences and that these sites are favorable for the invasion of mobile elements [31–33]. Many types of transposable elements (TEs) are described for teleostean fishes [34]. A possible role of TEs as a source of rRNA genes movement, which could generate the spreading of ribosomal clusters/copies, was visualized in fishes [35–37]. Hence, the organization of rRNA gene clusters seems to reflect their intense and particular evolutionary pathway and not the evolutionary history of the associated taxa in some fish group [36, 38]. Similar mechanisms might be responsible for the variation on the number and position of 18S rDNA sites observed in* Hypostomus*. The presence of adjacent heterochromatin on these sites may also facilitate genetic exchanges among non-homologous chromosomes, causing the dispersion of these sequences on the genome.

The species with 2*n* = 74 and 2*n* = 80 chromosomes (*H. albopunctatus*,* H. *aff.* paulinus,* and* H. topavae*) showed single chromosome pairs bearing 5S and 18S rDNA sites. However,* H. iheringii *(Regan, 1908) analyzed by Traldi et al. [26] showed a single pair bearing 5S rDNA and multiple pairs bearing 18S rDNA. Also, some species within this diploid number group have been reported with multiple NORs, such as *H*. aff.* agna *(2*n* = 74) [39],* H. nigromaculatus *(Schubart, 1964)  (2*n* = 76) [40], and* Hypostomus *sp. E (2*n* = 80) [40], therefore multiple 18S rDNA sites. In fact,* H. nigromaculatus *from the Lapa stream, São Paulo state, was reported to have 5S and 18S rDNA each on only one chromosome pair [22], while populations from Mogi-Guaçu and Tibagi Rivers showed multiple AgNORs, pointing to the existence of multiple 18 rDNA sites on such populations [40]. These results indicate that it is probable that this group of species also shows variations on the number and locations of rDNA sites among them. If it really is so, the whole group of species with 2*n* = 68 to 2*n* = 84 would be very heterogeneous regarding the number and location of the 18S rDNA sites, indicating that this character changed recurrently during the chromosomal diversification of the genus. All species in this diploid number range (2*n* = 74 to 2*n* = 84) analyzed until the present moment present a single pair bearing 5S rDNA sites. Further analyses are necessary to determine whether the increase of the diploid number is associated with the loss of 5S rDNA sites.

The presence of a centromeric/pericentromeric 5S rDNA site on the short arm of a metacentric/submetacentric pair is a particular feature of the 5S rDNA observed on most* Hypostomus* species analyzed on the present paper and also on other previously analyzed populations/species [20–22, 24, 25]. All species of* Hypostomus *analyzed so far but* H. cochliodon* have this 5S rDNA site on this particular location. Traldi et al. [22] consider the frequent occurrence of this site in this particular location a possible primitive condition for the genus. Possibly, the location of this site favored its permanence on the karyotype of most species, while telomeric sites seem to vary more easily. Further analyses are necessary to identify whether the lack of this particular 5S rDNA site occurs in other species of the* H. cochliodon *group or only in* H. cochliodon* or even on the studied population of this species.

Syntenic 5S and 18S rDNAs were observed in* H. commersoni*. The occurrence of syntenic 5S and 18S rDNAs is considered primitive for Loricariidae, with Trichomycteridae as outgroup [27]. This characteristic was already described for species of the subfamilies Neoplecostominae and Hypoptopomatinae [27] and in Hypostominae, for the tribes Ancistrini [29] and Hypostomini [22]. For Hypostomini, two species presented syntenic 5S and 18S rDNAs:* H. ancistroides *and* H. tapijara* Oyakawa, Akama, and Zanata, 2005 [22]. On the present study we presented one more species of Hypostomini (*H. commersoni*) that presented syntenic 5S and 18S rDNAs. All Hypostominae tribes that were analyzed through* in situ *hybridization with 5S and 18S rDNA probes until now present species with syntenic rDNAs. Although this condition may also be the primitive condition for Hypostominae, given its occurrence on different tribes, further studies are necessary to determine if it was maintained in* Hypostomus*. If* H. faveolus *really is a basal species for the genus, the possibility that the syntenic condition of the rDNA classes was lost early in the genus and then it reappeared due the apparent mobility of the rDNA on the genome must be considered as well.

The analysis here presented still includes a rather small number of species, considering the whole genus. However, we could observe a great variability on the number and location of the rDNA sites, reinforcing the hypothesis proposed by Artoni and Bertollo [41] that* Hypostomus *has a divergent chromosomal evolution when compared to other Hypostominae that tend to maintain more ancestral characteristics, such lower diploid numbers and one chromosome pair bearing AgNORs, therefore 18S rDNA. Further studies including more species, especially from northern basins, and analyses on the rDNAs and adjacent sequences would greatly contribute to clarify the dispersion mechanisms of these sequences within the genome of* Hypostomus*.

## Figures and Tables

**Table 1:** Species, collection sites, and number of analyzed specimens and catalog numbers (NUP) of the specimens deposited in the ichthyological collection of the Núcleo de Pesquisas em Limnologia, Ictiologia e Aquicultura.

Species	Males	Females	Locality	NUP
*Hypostomus albopunctatus *	3	3	Piquiri River, Nova Laranjeiras 24°56′54′′ S; 52°35′49′′ W	13532
*Hypostomus ancistroides *	4	11	Piquiri River, Nova Laranjeiras 24°56′54′′ S; 52°35′49′′ W	3902
*Hypostomus cochliodon *	4	1	Iguaçu River, Foz do Iguaçu 25°38′53′′ S; 54°27′28′′ W	13541
*Hypostomus commersoni *	0	1	Piquiri River, Nova Laranjeiras 24°56′54′′ S; 52°35′49′′ W	13540
	1	1	Iguaçu River, Foz do Iguaçu 25°38′53′′ S; 54°27′28′′ W	
*Hypostomus faveolus *	7	2	Taquaralzinho River, Barra do Garças 15°40′42′′ S; 52°17′52′′ W	13539
*Hypostomus hermanni *	5	4	Piquiri River, Nova Laranjeiras 24°56′54′′ S; 52°35′49′′ W	4927
*Hypostomus regani *	5	6	Piquiri River, Nova Laranjeiras 24°56′54′′ S; 52°35′49′′ W	13534
*Hypostomus *aff. *paulinus *	6	7	Piquiri River, Nova Laranjeiras 24°56′54′′ S; 52°35′49′′ W	13535
*Hypostomus strigaticeps *	8	7	Piquiri River, Nova Laranjeiras 24°56′54′′ S; 52°35′49′′ W	13536
*Hypostomus topavae *	9	6	Piquiri River, Nova Laranjeiras 24°56′54′′ S; 52°35′49′′ W	11430

**Table 2:** Available data regarding 5S/18S rDNA distribution in *Hypostomus*.

Species	2*n*	Karyotypic formula	5S	18S	Locality	Reference
*H. cochliodon *	64	12*m* + 16*sm* + 16*st* + 20*a*	Multiple	Simple	Paraná River basin	Present paper
*H. faveolus *	64	18*m* + 8*sm* + 22*st* + 16*a*	Multiple	Simple	Araguaia River basin	Present paper
*H. affinis *	66	14*m* + 14*sm* + 12*st* + 26*a*	Multiple	Multiple	Paraíba do Sul River basin	[20, 21]
*H. tapijara *	66	14*m* + 24*sm* + 14*st* + 14*a*	Multiple	Multiple	Ribeira do Iguape basin	[22]
*H. *prope* plecostomus *	68	12*m* + 16*sm* + 12*st* + 28*a*	—	Simple	Orinoco River basin	[23]
*H. ancistroides *	68	10*m* + 26*sm* + 32*a*	—	Multiple	Paranapanema River basin	[24]
*H. ancistroides *	68	14*m* + 14*sm* + 8*st* + 32*a*	Simple	Multiple	Paraná River basin	Present paper
*H. ancistroides *	68	14*m* + 16*sm* + 22*st* + 16*a*	Multiple	Multiple	Paranapanema River basin	[22]
*H. commersoni *	68	12*m* + 14*sm* + 14*st* + 28*a*	Multiple	Multiple	Paraná River basin (Iguaçu River)	Present paper
*H. commersoni *	68	12*m* + 14*sm* + 14*st* + 28*a*	Multiple	Multiple	Paraná River basin (Piquiri River)	Present paper
*H. hermanni *	72	10*m* + 8*sm* + 32*st* + 22*a*	Multiple	Multiple	Paraná River basin	Present paper
*H. regani *	72	8*m* + 16*sm* + 20*st* + 28*a*	Multiple	Simple	São Francisco River basin	[25]
*H. regani *	72	10*m* + 18*sm* + 44*st* − *a*	—	Multiple	Paranapanema River basin	[24]
*H. regani *	72	12*m* + 8*sm* + 10*st* + 42*a*	Simple	Multiple	Paraná River basin	Present paper
*H. strigaticeps *	72	10*m* + 16*sm* + 46*st* − *a*	—	Multiple	Paranapanema River basin	[24]
*H. strigaticeps *	72	12*m* + 12*sm* + 18*st* + 30*a*	Multiple	Multiple	Paraná River basin	Present paper
*H. albopunctatus *	74	8*m* + 14*sm* + 16*st* + 36*a*	Simple	Simple	Paraná River basin	Present paper
*H. paulinus *	74	10*m* + 12*sm* + 20*st* + 32*a*	Simple	Simple	Paraná River basin	Present paper
*H. paulinus *	76	6*m* + 16*sm* + 54*st* − *a*	—	Simple	Paranapanema River basin	[24]
*H. nigromaculatus *	76	12*m* + 22*sm* + 30*st* + 12*a*	Simple	Simple	Paranapanema River basin	[22]
*H. iheringii *	80	8*m* + 16*sm* + 28*st* + 28*a*	Simple	Multiple	Paranapanema River basin	[26]
*H. topavae *	80	14*m* + 10*sm* + 26*st* + 30*a*	Simple	Simple	Paraná River basin	Present paper
